# Development of *Cymbidium ensifolium* genic-SSR markers and their utility in genetic diversity and population structure analysis in cymbidiums

**DOI:** 10.1186/s12863-014-0124-5

**Published:** 2014-12-05

**Authors:** Xiaobai Li, Feng Jin, Liang Jin, Aaron Jackson, Cheng Huang, Kehu Li, Xiaoli Shu

**Affiliations:** Zhejiang Academy of Agricultural Sciences, Shiqiao Road 139, Hangzhou, 310021 People’s Republic of China; Hubei University, College of Life Sciences, Wuhan, 430062 People’s Republic of China; USDA-ARS, Dale Bumpers National Rice Research Center, Stuttgart, Arkansas 72160 USA; Agricultural Technology Extension Stations, Shaoxing County Agricultural Bureau, Shaoxing, 312000 Peoples Republic of China; School of Biological Sciences, The University of Hong Kong, Pokfulam Road, Hong Kong, SAR People’s Republic of China; State Key Lab of Rice Biology, International Atomic Energy Agency Collaborating Center, Zhejiang University, Hangzhou, 310029 Peoples Republic of China

**Keywords:** *Cymbidium ensifolium*, Genic-SSR, Genetic diversity, Population structure

## Abstract

**Background:**

*Cymbidium* is a genus of 68 species in the orchid family, with extremely high ornamental value. Marker-assisted selection has proven to be an effective strategy in accelerating plant breeding for many plant species. Analysis of cymbidiums genetic background by molecular markers can be of great value in assisting parental selection and breeding strategy design, however, in plants such as cymbidiums limited genomic resources exist. In order to obtain efficient markers, we deep sequenced the *C. ensifolium* transcriptome to identify simple sequence repeats derived from gene regions (genic-SSR).

**Result:**

The 7,936 genic-SSR markers were identified. A total of 80 genic-SSRs were selected, and primers were designed according to their flanking sequences. Of the 80 genic-SSR primer sets, 62 were amplified in *C. ensifolium* successfully, and 55 showed polymorphism when cross-tested among 9 *Cymbidium* species comprising 59 accessions. Unigenes containing the 62 genic-SSRs were searched against Non-redundant (Nr), Gene Ontology database (GO), eukaryotic orthologous groups (KOGs) and Kyoto Encyclopedia of Genes and Genomes (KEGG) database. The search resulted in 53 matching Nr sequences, of which 39 had GO terms, 18 were assigned to KOGs, and 15 were annotated with KEGG. Genetic diversity and population structure were analyzed based on 55 polymorphic genic-SSR data among 59 accessions. The genetic distance averaged 0.3911, ranging from 0.016 to 0.618. The polymorphic index content (PIC) of 55 polymorphic markers averaged 0.407, ranging from 0.033 to 0.863. A model-based clustering analysis revealed that five genetic groups existed in the collection. Accessions from the same species were typically grouped together; however, *C. goeringii* accessions did not always form a separate cluster, suggesting that *C. goeringii* accessions were polyphyletic.

**Conclusion:**

The genic-SSR identified in this study constitute a set of markers that can be applied across multiple Cymbidium species and used for the evaluation of genetic relationships as well as qualitative and quantitative trait mapping studies. Genic-SSR’s coupled with the functional annotations provided by the unigenes will aid in mapping candidate genes of specific function.

**Electronic supplementary material:**

The online version of this article (doi:10.1186/s12863-014-0124-5) contains supplementary material, which is available to authorized users.

## Background

*Cymbidium* is a genus of 68 species in the orchid family [1]. *Cymbidium* species are mainly distributed in the tropical and subtropical regions of Asia, including northwest India, China, Japan, Korea, the Malay Archipelago, and north and east Australia [2,3]. A total of 49 species can be found in China, including five famous species, i.e., *C. goeringii*, *C. faberi*, *C. ensifolium*, *C. kanran*, and *C. sinense*. These cymbidiums comprise some of the rarest plant species, with only a few surviving original populations and some reintroduced plants in the south of China, including Yunnan and Taiwan [4]. The fascinating varieties and shapes of their flowers endow these species with extremely high ornamental value that has attracted the world’s attention.

Knowledge of the genetic diversity and population structure of germplasm collections is an important foundation for plant improvement [5]. Estimation of genetic distance among germplasm is helpful in selecting parental combinations for creating segregating populations so as to maintain genetic diversity in a breeding program. However, genetic diversity may appear spatially structured at different scales, such as population, subpopulation or among neighboring individuals [6]. Population genetic analyses can provide important parameters including standing levels of genetic variation and the partitioning of this variability within/between populations [7]. The genetic diversity or population structure of *C. ensifolium* and other cymbidiums have been measured by using different molecular tools, including restriction enzyme polymorphism (RFLP) markers [3], random amplified polymorphic DNA (RAPD) markers [3,4,8], amplified fragment length polymorphism (AFLP) markers [4], polymorphisms of internal transcribed spacers (ITS) of nuclear ribosomal DNA and plastid, inter-simple sequence repeats (ISSR) markers [4,9], and SSRs [10,11]. Compared with RAPD, ISSR and ITS, SSR markers are more reliable, locus-specific, codominant, highly polymorphic, and well distributed throughout the genome [12]. Moreover, SSR’s only require polymerase chain reaction (PCR), which is a big advantage over RFLP and AFLP. These features make SSR’s well suited for marker-assisted selection, genetic diversity analysis, population genetic analysis, genetic mapping, and genetic map comparison in various species [13,14].

The number of SSR is very limited for *C. ensifolium*, due to limited sequence resources. Until now, the National Center for Biotechnology Information (NCBI) contained very limited *Cymbidium* sequence information, i.e., 692 nucleotide sequences and 78 expressed sequence tags (ESTs) (http://www.ncbi.nlm.nih.gov/nucest?term=cymbidium%5BOrganism%5D, verified 2014). RNA-seq provides a fast, cost-effective, and reliable approach for generating large-scale transcriptome data in non-model species, and also offers an opportunity to identify and develop genic-SSRs by transcriptome data mining [15]. Compared with traditional ‘anonymous’ SSRs from genomic DNA, these new genic-SSR markers have two advantages, i.e. a wealth of functional annotations and high transferability across taxa [15,16]. Herein, we extracted the total mRNA from *C. ensifolium* flower buds for RNA-seq, which resulted in 9.52 Gb of transcriptome data. From the *C.ensifloium* transcriptome, we obtained 55 new polymorphic microsatellite loci after testing their transferability across 59 *Cymbidium* accessions.

## Methods

### Plant materials

A total of 11 *C. ensifolium* accessions were employed to test genic-SSRs and additional 47 accessions from *C. lancifolium, C. floribundum, C. suavissimum, C. cyperifolium, C. qiubeiense, C. faberi, C. goeringii* and *C. sinense* were used to cross-test these markers among multiple species. The plants were grown and maintained in a greenhouse at the Zhejiang University under natural light (Table 1). Fresh leaf samples were collected from two or three seedling of each accession for genomic DNA extraction.

**Table 1:** **Fifty nine cymbidium accessions used for genetic analysis**

**Accession**	**Name**	**Group** ^**a**^	**Species**
1	Tiegusu	4	*C. ensifolium*
2	Qingshanyuquan	4	*C. ensifolium*
3	Jinsimawei	4	*C. ensifolium*
4	Jinhe	4	*C. ensifolium*
5	Yinsimawei	4	*C. ensifolium*
6	Dayibai	4	*C. ensifolium*
7	Dahongzhusha	2	*C. ensifolium*
8	Qiuhong	4	*C. ensifolium*
9	Baodao	4	*C. ensifolium*
10	Jinhe	2	*C. ensifolium*
11	Tianhe	4	*C. ensifolium*
12	Shisantaibao	4	*C. ensifolium*
13	TuerA	2	*C. lancifolium*
14	TuerB	2	*C. lancifolium*
17	DuohualanA	5	*C. floribundum*
18	GuoxianglanA	2	*C. suavissimum*
19	ShayelanA	1	*C. cyperifolium*
20	ShayelanB	1	*C. cyperifolium*
21	ShayelanC	1	*C. cyperifolium*
22	ShayelanD	1	*C. cyperifolium*
23	QiubeidonghuiA	2	*C. qiubeiense*
24	ShayelanE	1	*C. cyperifolium*
25	LvlanA	1	*C. faberi*
26	GuoxianglanB	5	*C. suavissimum*
27	lvlanB	1	*C. faberi*
28	DuohualanB	5	*C. floribundum*
29	Yuhudie	2	*C. goeringii*
30	Yinhe	5	*C. goeringii*
31	Silan	2	*C. goeringii*
32	Hexingmei	5	*C. goeringii*
33	Dasongmei	2	*C. goeringii*
34	Yipin	2	*C. goeringii*
35	Huangmei	2	*C. goeringii*
36	Puchunhong	2	*C. goeringii*
37	Chunjiansuxin	2	*C. goeringii*
38	Hongmeigui	2	*C. goeringii*
39	Wenyi	2	*C. goeringii*
40	Jiuxianmudan	2	*C. goeringii*
41	Dayipin	3	*C. faberi*
42	Ruyisu	2	*C. faberi*
43	Jiepeimei	3	*C. faberi*
44	Xinshanghaimei	3	*C. faberi*
45	Laoranzi	3	*C. faberi*
46	Xiashanjiujielan	3	*C. faberi*
47	Guifei	3	*C. faberi*
48	Mingyue	3	*C. faberi*
49	Xiyang (Qingxiang)	3	*C. faberi*
50	Yuchan	3	*C. faberi*
51	QiubeidonghuiB	2	*C. qiubeiense*
52	DuohualanC	5	*C. floribundum*
53	DuohualanD	5	*C. floribundum*
54	QiubeidonghuiC	2	*C. qiubeiense*
57	Wuzicui	2	*C. sinense*
58	Jinhuashan	2	*C. sinense*
59	Rixiang	2	*C. sinense*
60	Qihei	2	*C. sinense*
61	Damo	2	*C. sinense*
62	Hongmeiren	2	*C. sinense*
63	Baimo	2	*C. sinense*

^a^Five groups indicated by population structure analysis.

### Genic-SSR search and primer design

Total RNA was isolated from native cultivar of *C. ensifolium* “Tiegusu” using TRIzol® reagent (Invitrogen, CA, USA) and treated with RNase-free DNase I (TaKaRa Bio, Dalian, China) for 45 min according to the manufacturer’s protocol. The RNA was used in cDNA library construction and Illumina deep sequencing [17]. The raw sequencing reads were stringently filtered, and high-quality reads were assembled de novo using Trinity with an optimized k-mer length of 25 [18]. MSATCOMMANDER V. 0.8.2 [19] was used to analyze SSR distribution. The minimum number of repeats for SSR detection was as follows: six for di-SSRs, and four for tri-, tetra-, penta-, and hexa-SSRs. The open reading frame (ORF) and untranslated region (UTR) within unigenes were identified using Trinity [18]. Software Primer3.0 [20] was used to design primers for genic-SSR loci with sufficient flanking sequences.

Unigenes containing genic-SSRs were compared with protein databases, including the non-redundant (Nr) database (http://www.ncbi.nlm.nih.gov/), using BLASTX with a significance cut-off *E*-value of 1e^−5^ [17]. For the non-redundant annotations, BLAST2GO V. 2.4.4 was used to obtain Gene Ontology (GO) annotations of unique transcripts [21]. Metabolic pathway analysis were performed based on the pathways of *Oryza sativa* in the Kyoto Encyclopedia of Genes and Genomes (KEGG) [22,23]. The unigene sequences were also aligned to the KOG (Eukaryotic Orthologous Groups) database to predict and classify possible functions [24].

### Genotyping

Genomic DNA was extracted from leaf samples as previously described [25]. PCR primers were synthesized by Life Technologies (AB & Invitrogen, Shanghai, China). PCR reactions were conducted based on a previously published protocol [26]. The PCR products were separated through polyacrylamide gel electrophoresis using 8% bis-acrylamide, 0.5% TBE buffer, 0.07% APS, and 0.035% TEMED. The gel was run at constant 120 V for approximately 3 h in 1× TBE buffer. The gel was silver-stained according to Li’s procedure [27], and was then documented using a scanner. The genotype was determined by analysis of the bands’ pattern, dependent on the number and the position of bands.

### Statistical analysis

Genetic distance was calculated using Nei’s distance [28]. Phylogenetic reconstruction was based on the unweighted pair-group method that utilizes the arithmetic average (UPGMA) method implemented in PowerMarker version 2.7 [29]. The tree that was used to visualize the phylogenetic distribution of accessions and ancestry groups was constructed using MEGA version 4 [30]. A model-based program structure [31] was used to infer population structure with 5,000 burn-in and run length. The model allowed for admixture and correlated allele frequencies. The number of groups (K) was set from 1 to 10, each with 10 independent runs. The most probable structure number (K) was determined through log probability [32]. Principal component analysis (PCA), which summarizes the major patterns of variation in a multi-locus data set, was performed using NTSYSpc version 2.11 V [33]. Two principal components were used to represent the dispersion of the collection accessions graphically [34]. PowerMarker was used to calculate the average number of marker alleles and the polymorphism information content (PIC) values. Fixation index (*Fst*), which indicates the differentiation among genetic groups, was calculated using an Analysis of Molecular Variance (AMOVA) approach in Arlequin V2.000 [35].

## Results

### Genic-SSR search and primer design

In *C. ensifolium* transcriptome, 98,819,349 reads, (9.52 Gb), were obtained after removal of adaptor sequences, ambiguous reads, and low-quality reads (Q-value <25). These reads were used for the subsequent assembly, and then resulted in 101,423 unigenes (139,385,689 residues). The length of unigenes averaged 1,374 bp and ranged from 351 bp to 17,260 bp. The data were uploaded to the NCBI (http://orchidbase.itps.ncku.edu.tw/est/home2012.aspx) for public use (Accession: SRA098864).

In the present study, 7,936 genic-SSRs were identified, with one SSR locus for every 17.56 kb (kb/SSR). Estimated locations (coding, 5′UTR or 3′UTR) were obtained for 5,524 genic-SSRs. Sequence information could not be determined for the remaining 2,412 genic-SSR regions, because the locations were extended over both estimated coding and non-coding regions. Given such high numbers of SSR, we analyzed the sequence data to isolate high quality SSR loci for further testing. An important factor considered was the locations of SSRs relative to ORFs. SSRs within UTR are exposed to lower selective pressure than those in coding regions and have a higher likelihood of being polymorphic [36]. Another two factors are the length of the motif and the number of the repeat motif, which are often associated with polymorphism [37]. Thus, SSR’s within UTR, with short motifs and high repeat number would be the best marker candidates. Herein, we selected 80 genic-SSRs and designed primers based on their motifs, sizes and locations.

### Genic-SSRs’ profile

All primer sets were initially tested among 12 *C. ensiflolium* accessions, and then were cross-tested among other 47 *Cymbidium* accessions (Table 1). Of the 80 genic-SSR primers, 62 amplified within *C. ensifolium* accessions successfully, and 55 showed polymorphism when cross-tested among all 9 cymbidium species (Additional file 1: Figure S1). These accessions belonged to 9 cymbidium species i.e. *C. ensifolium, C. lancifolium, C. suavissimum, C. cyperifolium, C. qiubeiense, C. floribundum, C. goeringii, C. faberi and C. sinense*. Among the 55 polymorphic markers, the PIC averaged 0.407, ranging from 0.033 (for both SSR29 and SSR31) to 0.863 (for SSR73). Similarly, allele number averaged 5.75, ranging from 2 (for SSR06, SSR24, SSR29, SSR31, SSR46, SSR55, SSR71, SSR75 and SSR79) to 16 (for SSR73) (Table 2). These results suggested that genic-SSR markers had a broad applicability within *Cymbidium* genus.

**Table 2:** **List of the 62** ***C. ensifolium*** **genic-SSR primers including their unigenes’ annotation**

**Name**	**Product size (bp)**	**SSR**	**SSR location**	**Primer**	**Homologs in non-redundant database (accession in Genebank)**	**GO annotation**	**KOG annotation**	**KEGG annotation**	**Allele number**	**PIC**
SSR01	400-500	(AC)8	utr5	F: AACGCCATGTCCAATACCC	PREDICTED: probable transcription factor KAN2-like (XP_002278005.2)	GO: 0003677	KOG1601	NULL	5	0.552
				R: GGAGGGCTTATTTGCAGCG						
SSR02	300-400	(AC)8	utr5	F: CTCCTTCAAGCTTCTGCCC	PREDICTED: histone-lysine N-methyltransferase, H3 lysine-9, H3 lysine-27, H4 lysine-20 and cytosine specific SUVH2 (XP_002282386.1)	GO: 0042393	NULL	NULL	NA	NA
				R: GACCGCAGCGTTAATGACC						
SSR03	400-500	(AC)8	utr3	F: CTCGGTTCATTTGCAGCCC	PREDICTED: mitochondrial import receptor subunit TOM20 (XP_002269795.1)	GO: 0045040	NULL	NULL	7	0.690
				R: GGGTGGGTATGGCGAAATC						
SSR04	400-500	(AC)8	utr3	F: AGAATCTGCCAACCCTTGATAC	NULL	NULL	NULL	NULL	6	0.657
				R: GCAGATGCCAGTTAGAATGGG						
SSR05	1000	(AC)8	utr3	F: AGAACTGCAGGTGTGAAGC	PREDICTED: protein CbbY, chromosomal-like isoform 1 (XP_003574671.1)	GO: 0016787	NULL	NULL	3	0.125
				R: GGCTTGAAGTGGCGATAACC						
SSR06	600	(AC)9	utr3	F: GCGTCTGCTGAAACGATGG	Putative steroid 22-alpha-hydroxylase (AAN60994.1)	GO: 0016020	KOG0157	K09587	2	0.063
				R: AAACAGCGCCTGTCATTCC						
SSR07	300-400	(AC)9	utr3	F: ACGCTGCATCCCATTTCAC	PREDICTED: uncharacterized protein LOC100243361 (XP_002276849.2)	GO: 0008987	NULL	K03517	4	0.180
				R: CAGTCTGTTGAGGAAGCCG						
SSR08	100-200	(AC)10	utr3	F: TGCTGGAATACATGCGAGAC	Predicted protein (XP_002298559.1)	GO: 0023014	KOG0610		14	0.753
				R: GTTTGCCGAAGCCAGTGC						
SSR11	600	(AG)10	utr3	F: AACTGACAAGCATCTGCAAG	Uncharacterized protein LOC100273319 precursor (NP_001141232.1)	GO: 0005774	NULL	NULL	6	0.477
				R: CTGCTGCATTGGCCTTACC						
SSR12	300	(AG)11	utr5	F: TCAGCCGAGGTTAGTATACGG	PREDICTED: phosphatidylinositol-4-phosphate 5-kinase 9-like (XP_002265706.1)	GO: 0016020	KOG0229	K00889	NA	NA
				R: CTTGCCATCTCAGCAGTCG						
SSR13	400-500	(AG)11	utr5	F: GCTGCTGCTTGGTGGAAAC	Predicted protein (XP_002317724.1)	GO: 0005488	NULL	NULL	6	0.343
				R: GCGCTCGTTGTATGGCTTG						
SSR14	300	(AG)11	utr5	F: CACAGCAGCTCACAATCCTG	Unnamed protein product (CBI20568.3)	GO: 0006099	KOG1257	K00029	8	0.467
				R: TACAGCCCTGTTTACCGCC						
SSR15	100-200	(AG)11	utr3	F: CCTTCTCTCCGCGTACCAG	PREDICTED: uncharacterized protein LOC100825549 (XP_003558805.1)	GO: 0005783	NULL	NULL	4	0.339
				R: CTTCGGTTGGCGTTTAGGG						
SSR16	300-400	(AG)11	utr5	F: GCCCACAGCAATCCATCTG	PE repeat family protein (XP_003014087.1)	NULL	NULL	NULL	7	0.348
				R: GCAGTCGAAGAAACCGTGG						
SSR17	400	(AG)11	utr5	F: GGATCACCAACAGCATGGG	Transcription factor (ADG57844.1)	GO: 0003677	NULL	K09060	4	0.417
				R: TCCACCAAGAGCAAGGATG						
SSR18	300	(AG)11	utr5	F: TGAAACGGTTGGCTCTAGTTC	Conserved hypothetical protein (XP_002527260.1)	NULL	NULL	NULL	13	0.519
				R: AGCAAGCACTGACCTGAAAC						
SSR21	300-500	(GT)8	utr3	F: TGGGCGACAGATCGAGTTC	Hypothetical protein OsJ_08996 (EAZ25197.1)	NULL	NULL	NULL	15	0.794
				R: ACATGGACCACAGCATTCC						
SSR22	200-300	(GT)9	utr3	F: TATGCGTCTCTCCCAACCG	14-3-3-like protein B-like(ACQ45020.1)	GO: 0019904	KOG0841	K06630	10	0.572
				R: AAGCTAGTGGCCTTTGGTG						
SSR23	100-200	(GT)10	utr3	F: CGGCGATCGATTTATGAGCC	PREDICTED: beta-amylase 1, chloroplastic isoform 1 (XP_002285569.1)	GO: 0005634	NULL	K01177	NA	NA
				R: CGATACTCCTCAATGTCGTGG						
SSR24	200-300	(GT)11	utr5	F: TCGGTAACCTGTTGCAAGG	PREDICTED: flavin-containing monooxygenase YUCCA6-like (XP_003550114.1)	GO: 0050661	NULL	K11816	2	0.063
				R: ACCTGTGAAGCTACCAGAC						
SSR25	100-250	(GT)11	utr3	F: GAATCTCTCGCACCCGAAG	Aspartyl/glutamyl-tRNA(Asn/Gln) amidotransferase subunit B, putative (XP_002528338.1)	GO: 0006536	NULL	K02434	NA	NA
				R: TGGACAACATCAAGTGACGC						
SSR26	100-250	(AAG)7	utr3	F: GCTTTATGCGACATCTGCG	Unnamed protein product (CBI25980.3)	GO: 0005634	KOG1901	NULL	11	0.638
				R: CGTCGGTTCCATGCACATC						
SSR27	500-600	(AGC)5	utr3	F: CTGCCTTCACAGCTAATGCC	Os04g0512400 (NP_001053298.1)	GO: 0046872	NULL	NULL	3	0.313
				R: GCATGCTTGGACGCTGAAC						
SSR29	200-300	(AGC)6	utr3	F: AGCAAACGGCAAGTCATGG	RING finger protein 113A, putative (XP_002522169.1)	GO: 0016020	NULL	K13127	2	0.033
				R: ATTCGACTACCAGCCGGAC						
SSR30	200-300	(AGG)5	utr3	F: AAACGAAGGGCTGGAAGTC	NULL	NULL	NULL	NULL	9	0.486
				R: TTTGACATCGGGAAGTGGC						
SSR31	100-200	(AGG)5	utr5	F: GGGATGCATAGACCTTTCGC	Protein MSF1, putative (XP_002535293.1)	GO: 0005739	KOG3336	NULL	2	0.033
				R: CAGGTTCAACGGCATCGTG						
SSR32	1000-1100	(AGG)5	utr3	F: CTCCGGCCTCTGGTTACTC	PREDICTED: HVA22-like protein j (XP_002281038.1)	NULL	KOG1726	NULL	7	0.601
				R: AGTGATGAGGCTTGGACCG						
SSR34	700-900	(AGG)6	utr5	F: GAGAGGGAATTGCAGTGGC	Hypothetical protein (BAI68347.1)	NULL	NULL	NULL	6	0.696
				R: ACCGAGCTAGCACTTCATC						
SSR35	700-900	(ATC)5	utr5	F: AGAGTGATTGTCCAGCTCCG	PREDICTED: diacylglycerol kinase-like (XP_003534537.1)	GO: 0009395	NULL	K07029	4	0.475
				R: TGCCTCTCTGGTGATGTCC						
SSR36	400-500	(ATC)5	utr3	F: AGTATTGGACCCTCCAGGC	NULL	NULL	NULL	NULL	5	0.536
				R: AGAGGATCATGGTGTTAGGC						
SSR37	200-300	(ATC)5	utr5	F: GGCCTAGCCAGCCCTTC	NULL	NULL	NULL	NULL	3	0.205
				R: ATTTGGATCGCACAAGCGG						
SSR38	200-300	(ATC)6	utr3	F: TAGCCCATGCCAGTGTTCC	LOC100285373 (NP_001151738.1)	GO: 0007165	NULL	NULL	3	0.149
				R: AACTGCCACAAGAGAAGGC						
SSR39	1000-1100	(ATC)6	utr3	F: ACAGACTGCCACCTGTTCC	unnamed protein product (CBI38283.3)	GO: 0008234	KOG1870	K11835	5	0.401
				R: GCCTGCCTTTGCTCCTTG						
SSR40	400	(ATC)6	utr5	F: ACAAGCATCATCCCAAATTCC	PREDICTED: probably inactive leucine-rich repeat receptor-like protein kinase At2g25790-like (XP_002267653.1)	GO: 0007165	NULL	NULL	NA	NA
				R: GCAGAAACTGGAGCTTGCC						
SSR42	200-300	(CCG)5	utr5	F: GACGACATATCGCGTTCGG	unnamed protein product (CBI18667.3)	GO: 0003779	KOG0160	NULL	6	0.564
				R: CTCAGCCACACCCAAGAGG						
SSR43	500	(CCG)5	utr5	F: GGAGCTGCATACGCAAGTG	glycinebetaine/proline transporter (BAJ07206.1)	GO: 0015193	NULL	NULL	7	0.572
				R: AGCTTCTCACTGCCTCCAG						
SSR44	300-400	(CCG)5	utr5	F: CGTCGACTCCTCGAGATCC	predicted protein (BAJ93650.1)	GO: 0046872	NULL	NULL	NA	NA
				R: GCGTTAGCAGCAGTCTTGG						
SSR45	400-500	(CCG)5	utr5	F: GCCTTACACATCCCTTCCAAC	unnamed protein product (CBI33381.3)	GO: 0005515	KOG0550	NULL	5	0.338
				R: TGCCTGCTGATAGTTTGCC						
SSR46	200-300	(CCG)6	utr5	F: CCTTCGTGGACTCAACAGC	hypothetical protein SORBIDRAFT_01g031510 (XP_002465065.1)	NULL	NULL	NULL	2	0.063
				R: TCTCGTGCAGGAATCGGTC						
SSR47	400-500	(CCG)6	utr3	F: GCAGGTGTCCTCATCGGAG	CONSTANS-like protein (ADN97077.1)	GO: 0005622	KOG1601	NULL	NA	NA
				R: CTCCGGCTAACTCCATCCC						
SSR49	300	(CCG)7	utr3	F: AGAGGGCCACCTGCTTTC	predicted protein (XP_002312577.1)	NULL	KOG1863	NULL	6	0.549
				R: GCCAATTGCCAGATGGACG						
SSR52	400-500	(CCT)4	utr5	F: AAGAGGCACTGCAAGACCC	hypothetical protein SORBIDRAFT_01g031070 (XP_002465040.1)	NULL	NULL	NULL	8	0.378
				R: CGTTCCAGCAACCCATAGC						
SSR53	100-200	(CCT)4	utr5	F: GCTGAAGGTTCCGGTCCTC	PREDICTED: uncharacterized protein LOC100830480 (XP_003580351.1)	NULL	NULL	NULL	8	0.700
				R: TCCGCCTCTTTAAGCCGAC						
SSR54	200-300	(CCT)4	utr5	F: ATCTTCCCTCCACATCGGC	hypothetical protein MTR_1g083540 (XP_003591171.1)	GO: 0005886	NULL	NULL	5	0.422
				R: TGGAGAAGAGTCGACCAGC						
SSR55	200-300	(CCT)4	utr5	F: TGGAATGGTTCTAGGGCTTC	hypothetical protein (CCA65980.1)	NULL	NULL	NULL	2	0.323
				R: CCACTGGTACCCTCCTTGG						
SSR56	900-1000	(CCT)5	utr5	F: TGCTTCATTGTTGGAGGCG	predicted protein (XP_002324427.1)	GO: 0008643	KOG0254	NULL	5	0.315
				R: AGTGGACGGAGAGTCAAGC						
SSR59	200-300	(CGG)5	utr3	F: GTTTCCAACGGTCAGCTCG	leucine-rich repeat transmembrane protein kinase family protein (NP_177007.1)	GO: 0005524	NULL	NULL	3	0.432
				R: GTGATGTGGTAGCATCGCC						
SSR60	200-300	(CGG)5	utr5	F: TACGGTTTCGACCAGCCTC	Unnamed protein product (CBI41056.3)	GO: 0005634	KOG0265	K10143	4	0.203
				R: CCATGCAGATCGGGCAAAG						
SSR62	300-400	(CGG)6	utr5	F: GGTGGGTTAGACCAGCTCC	Hypothetical protein OsI_29809 (EAZ07555.1)	GO: 0005634	NULL	NULL	6	0.570
				R: TCCTCAAGGCAAAGCTCCC						
SSR63	100-200	(CGG)6	utr5	F: CTTCCTCCACCTGGATCGC	Uncharacterized protein LOC100277474(NP_001144494.1)	GO: 0008270	NULL	NULL	4	0.308
				R: CTGCCGATCAATCCGAGAC						
SSR64	400-500	(CGG)6	utr3	F: CGCTCAAAGAGATGGCACG	Os01g0226200 (NP_001042462.1)	NULL	NULL	NULL	11	0.627
				R: TAGTACGGCGCTGCTTGAG						
SSR66	300-400	(CGG)7	utr3	F: CATCTTCCTTGCCCGATGC	PREDICTED: pentatricopeptide repeat-containing protein At5g42310, mitochondrial (XP_002272226.1)	NULL	KOG4197	NULL	4	0.126
				R: CCCGCCAAATTTCGAGACC						
SSR68	100-200	(GAT)5	utr3	F: CCAGATCGAATGGCTACGC	Hypothetical protein VITISV_010525 (CAN79523.1)	GO: 0003723	NULL	NULL	4	0.211
				R: CAAGGAGCTCGTCGAAGG						
SSR69	200-300	(GAT)5	utr5	F: GTTTAGGCTAGCAGTGCGG	NULL	NULL	NULL	NULL	3	0.149
				R: TGAGAACGTAGTGAAGTTGCC						
SSR70	200-300	(GAT)7	utr3	F: CCCAACGCAGAACGATAGC	NULL	NULL	NULL	NULL	5	0.529
				R: CGGTGGCACAAATGGAACG						
SSR71	400-500	(GGT)5	utr5	F: GCATCGAAACCACTGTCGC	Hypothetical protein SORBIDRAFT_09g018170 (XP_002439663.1)	NULL	NULL	NULL	2	0.262
				R: CCCTAGCCGGAGTCTCAAC						
SSR73	100-200	(GGT)5	utr3	F: GGACACAATGGAGACGAAGG	T4.15 (CCH50976.1)	GO: 0044238	NULL	NULL	16	0.863
				R: TGCATGAAACCACATGGC						
SSR75	400-500	(GTT)6	utr3	F: GCCTTTGACCATTCCGTGC	Mitogen-activated protein kinase 1 (AEQ28763.1)	GO: 0043622	KOG0660	K04371	2	0.118
				R: GGCCGCCATGAGTAAGAAC						
SSR76	500-600	(GTT)6	utr5	F: AGACAGAGAGTCCCTAAAGGC	NULL	NULL	NULL	NULL	7	0.519
				R: CAGGGATGTTAAGTGGGCTG						
SSR77	300-400	(GTT)6	utr3	F: TTTGTGGCAGTGGAAAGCG	NULL	NULL	NULL	NULL	5	0.470
				R: TGATACCAATGGCAAGGCG						
SSR79	200-300	(GTT)6	utr5	F: AGGATTCATGTAGCCGACCTC	Hypothetical protein OsI_35425 (EEC67831.1)	NULL	NULL	K10728	2	0.207
				R: TCCCTGAAGGAGGCAAACC						
SSR80	400-500	(GTT)7	utr3	F: GCACCCAGCTTGTTTGAGG	NULL	NULL	NULL	NULL	8	0.626
				R: CCCATACATTACAGGCAAGC						

Note: A total of 62 genic-SSR markers successfully amplified were listed, however 55 polymorphic markers were used in subsequent population analysis or cross species comparison. NULL: no annotation. NA: monomorphic marker.

### Genetic diversity and population structure

**Figure 1:**
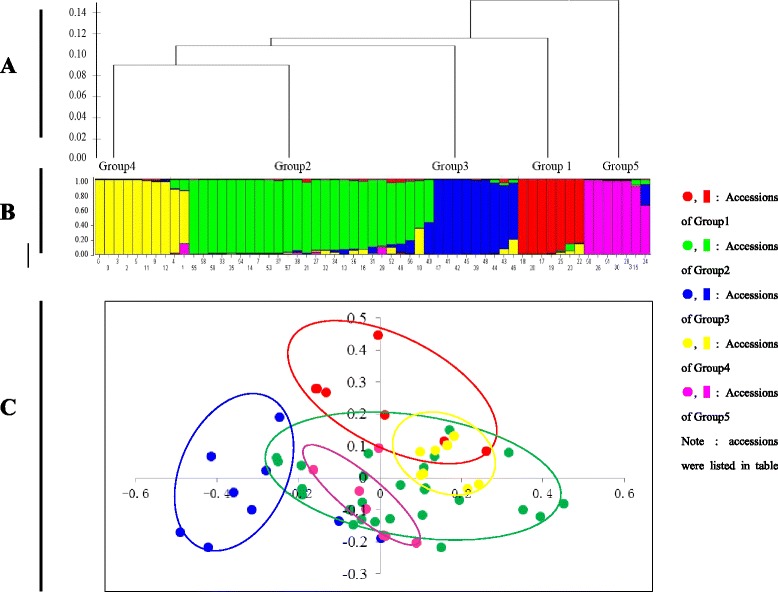
**Population structure based on 55 polymorphic genic-SSRs. A**: Phylogenetic tree of five main groups, **B**: The estimated group structure with each individual represented by a horizontal bar, and **C**: PCA of five main groups. Group 1 in red, Group 2 in green, Group 3 in blue, Group 4 in yellow, Group 5 in purple, and admixed cultivars indicated by a slight blue gradient.

These genic-SSRs revealed genetic variation among accessions. The genetic distance among accessions ranged from 0.016 to 0.618, with an average of 0.391. The model-based clustering method revealed five groups (Figure 1A and B). Group 2 had the most accessions (26), with the highest mean genetic distance (MGD) of 0.431 among these accessions; Group 4 had 10, with an average distance of 0.236; Group 5 had 7, with MGD of 0.332; Group 1 and Group5 both had 7 accessions, with MGD of 0.155 and 0.332, respectively; Group 3 had 9, with MGD of 0.213. Genetic distance among five groups was from 0.340 (between group 1 and group 5) to 0.176 (between group 2 and group 4, with average of 0.248) (Table 3).

**Table 3:** **Pairwise comparison of Nei’s genetic distance among groups and mean of genetic distance within group based on 55 polymorphic genic-SSRs**

**Group**	**No. of accessions**	**Mean genetic distance (MGD)**	**Group 1**	**Group 2**	**Group 3**	**Group 4**	**Group 5**
Group 1	7	0.155	0.000				
Group 2	26	0.430	0.217	0.000			
Group 3	9	0.213	0.252	0.190	0.000		
Group 4	10	0.236	0.212	0.176	0.234	0.000	
Group 5	7	0.332	0.340	0.263	0.298	0.293	0.0000

**Figure 2:**
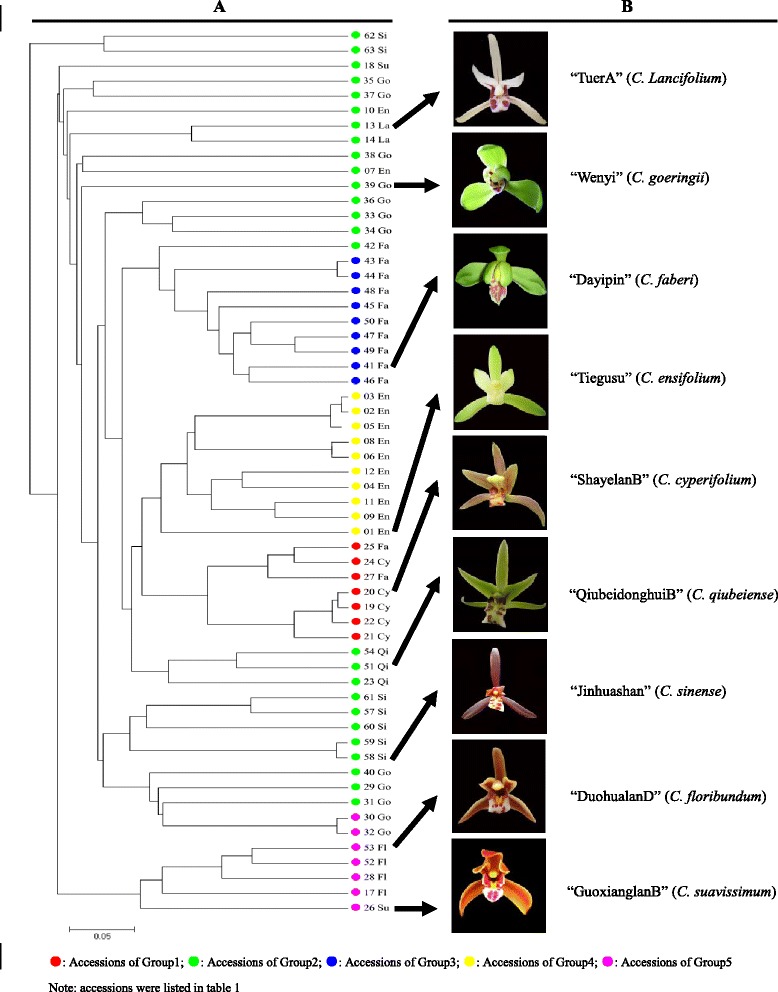
**Phylogenetic tree of cymbidiums. A**: Unweighted pair-group method tree of 59 accessions based on 55 polymorphic SSRs, and **B**: Morphology of cymbidium’s flower. Group 1 indicated by red dot, Group 2 by green dot, Group 3 by blue dot, Group 4 by yellow dot, Group 5 by purple dot.

The five groups revealed by the model-based clustering analysis consisted of different species. Three groups comprised more than one species, whereas the other two only comprised one species. Group 1 included two species i.e. *C. cyperifolium* and *C. goeringii*; Group 2 included *C.ensifolium*, *C. lancifolium*, *C. suavissimum*, *C. qiubeiense*, *C. goeringii*, *C. faberi*, and *C. sinense*; Group 5 included *C. floribundum*, *C. suavissimum* and *C. goeringii*. Goup 3 and Group 4 included only *C. faberi* and *C.ensifolium*, respectively (Figure 2).

The first two components in PCA (47.87% and 21.59% of total variation, respectively) discriminated the five groups at a certain level. Basically, accessions in group 1 and group 3 stayed alone, whereas group 2 overlapped with group 4 and group 5 (Figure 1C). In the phylogenetic tree, group 2 and group 4 were genetically close, while group 5 was relatively distant from the other groups (Figure 1A). In addition, a few accessions in group 2 had admixture ancestry from group 3 and group 4, while accessions in group 3 and group 1 had less admixture ancestry (Figure 1B). AMOVA results showed that 25.34% of the total variation was among groups, while 74.66% of the variation was within groups. The *F*_*ST*_ was 0.25, as indicated by the AMOVA approach.

### Genic-SSR annotation

**Figure 3:**
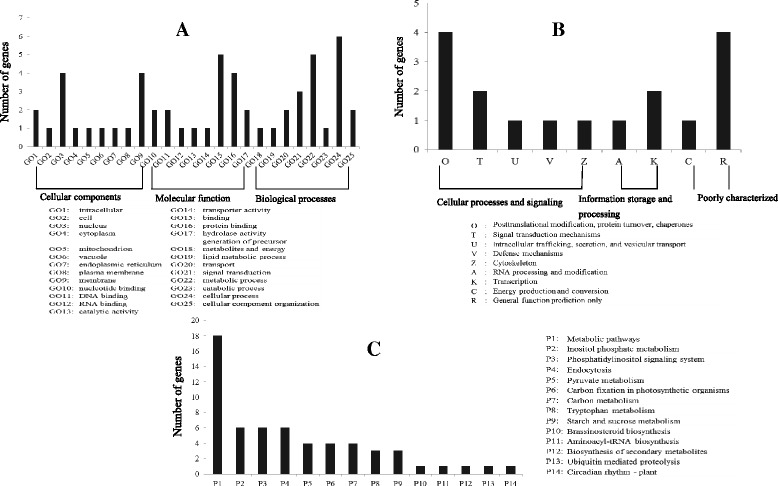
**Functional annotations of unigenes containing SSR. A**: KOG prediction and possible function, **B**: GO functional classifications, and **C**: KEGG pathways involved.

Annotations of these unigenes provide biological information for 62 genic-SSRs, such as KOG clusters, GO, and KEGG pathway information. Distinct gene sequences were first searched using BLASTX against the Nr database. The results showed that 53 unigenes had hits that exceeded the E-value threshold. In the present study, 39 unigenes were categorized into 25 GO terms in three GO ontologies (Figure 3A). Two groups “membrane” and “nucleus”, one group “binding”, and one group “cellular process” comprised the most representative genes found in cellular components, molecular function, and biological processes, respectively. Out of 53 hits in the Nr databases, 18 sequences were classified into 9 KOG categories (Figure 3B). Among the 9 KOG categories, “General function prediction only” and “Posttranslational modification, protein turnover, chaperones” were the two largest groups. When referenced to rice (*Oryza sativa*), 15 unigenes were found to be involved in 14 pathways (Figure 3C). The most highly representative one was “metabolic pathways”, where unigenes shared similarity with 18 rice sequences.

## Discussion

### Diversity

Because genic-SSR markers are derived from transcribed regions of DNA, they are expected to be more conserved and have a higher rate of transferability than anonymous SSR markers [38]. Herein, 55 *C. ensifolium* polymorphic genic-SSR markers exhibited 100% transferability across the 59 accessions of the 9 *Cymbidium* species tested. It is common that genic-SSRs possess a high potential for inter-specific transferability [39,40]. Other markers such as RAPD’s, ISSR’s and non-genic SSR’s have also been used with success among *C. ensifolium* and the *Cymbidium* species reflecting the genetic similarity among many members of the genus [8,11,15].

The conserved nature of the genic-SSRs may limit their polymorphism relative to randomly selected SSR’s. In this study, PIC of genic-SSR markers averaged 0.407, lower than 0.782 [5] and 0.639 [11] of anonymous SSR’s tested on Chinese cymbidiums in other studies. The pair-wise genetic distance averaged 0.391 among 59 accessions, which is also lower than that from previous studies conducted on Chinese Cymbidiums using other molecular markers [3,8,41-44]. Even though genic-SSRs revealed less variability than SSRs, these markers still reveal sufficient levels of variation for population genetic analysis.

### Population structure

One of the biggest advantages for genic-SSRs is that they allow one to make direct comparisons among taxa without running the risk that locus-specific differences might mask true species-level differences, such as overall levels of genetic diversity, the extent of population structure, and so on. However, the greatest concern with the utilization of genic-SSRs in genetic studies is that selection on these loci might influence the estimation of population genetic parameters. While a recent study by Woodhead et al. [45] revealed that estimates of population differentiation based on genic-SSRs are comparable to those based on both SSRs and AFLPs in ferns, and large-scale comparative analysis suggest that only a very small percentage of all genes has experienced positive selection [46,47], a small fraction of SSRs will be inevitably subject to selection. The view is consistent with the theory that most mutations are neutral, or nearly neutral, [48] or, at least, do not change the function of gene products appreciably [49].

In the population genetic analysis, almost all accessions from the same species clustered together. *C. suavissimum* and *C. floribundum* were clustered into one brand, and clearly distinguished from other cymbidiums. Two of them belong to Section *Floribundum*, and have a distant relationship with other cymbidiums. However, the genetic relationship between C. *goeringii* and *C. sinensis* was close, which was congruent with the previous reports [5,11]. The close relationship was also found between *C. ensifolium* and *C. cyperifolium*. In the intersection level, we discovered that two accessions of *C. faberi* were clustered with *C. cyperifoliumm*, and accessions of *C. lancifolium* and *C. ensifolium* were scattered among ones of *C. goeringii*. The splitting feature of these clusters might be linked to the non-homologous synapomorphy, even though accessions belonged to different species. The accessions of *C. goeringii* did not always form a separate cluster in the phylogenetic tree or were not grouped together in structure analysis, suggesting that they were polyphyletic. Previous morphologic, cytogenetic, and molecular studies have shown that the major lineages of Chinese cymbidiums are ambiguous. *C. ensifolium* and *C. sinense* are classified in section *Jensoa*; *C. faberi* and *C. goeringii*, are classified in section *Maxillarianthe*; *C. faberi*, *C. kanran*, and *C. longibracteatum* are classified in one group; *C. ensifolium*, *C. goeringii*, and *C. sinense* are categorized into another group [44].

### Genic-SSR annotation

Putative functions were assigned to those unigenes containing SSRs by sequence similarities. These unigenes were involved in a wide range of functions, which indicated that these genic-SSRs were likely important biologically characters. For example, unigene containing SSR47 shares homology with CONSTANS-like protein. In Arabidopsis, the CO (CONSTANS) gene has an important role in the regulation of flowering by photoperiod [50]. Unigene containing SSR43 has homology with a glycinebetaine/proline transporter. The accumulation of glycinebetaine (GB) is one of the adaptive strategies to adverse salt stress conditions [51]. The transporters mediate the uptake of GB and/or proline in many plant species e.g. *Arabidopsis thaliana* [52], tomato (*Solanum lycopersicum*) [53], rice (*Orazy sativa*) [54], barley [55]. Unigene having SSR75, was annotated as mitogen-activated protein kinase (MAPK). MAPK cascades function as key signal transducers that use protein phosphorylation/dephosphorylation cycles to channel information [56]. In the plant, MAPKs have been shown to regulate numerous cellular processes, including biotic stress relief [57,58]. Although some unigenes with SSRs had no match to known genes in current gene database, they will likely gain functional annotations as the knowledge of plant genes increases. Compared with anonymous SSRs, genic-SSR markers have a higher probability of being functionally associated with differences in gene expression, which may be in identifying associations between genotype and phenotype. Mapping of genic-SSRs will also provide a map location, in many cases, for genes with known functions.

## Conclusion

In this work, 7,936 genic-SSRs were identified in *C. ensifolium* transcriptome and their characterizations were further analyzed. A total of 80 genic-SSRs were chosen for validation, and 55 markers successfully yielded polymorphism across 9 *Cymbidium* species including 59 accessions. The high transferability of genic-SSR will be a powerful resource for molecular taxonomic studies and construction of a reference molecular map of the *Cymbidium* genome. Since genic-SSR markers belong to gene-rich regions of the genome, some of these can be exploited for use in marker-assisted breeding of *Cymbidium*. Therefore, the set of genic-SSR markers developed here is a promising genomic resource.

## Additional file

Additional file 1: Figure S1. Polyacrylamide gel electrophoresis profile of SSR62 a and SSR73 b. M: Maker DL2000; 1–63: cymbidium accession listed in Table 1.
