# Supplementary figures and images for: Development of Cymbidium ensifolium genic-SSR markers and their utility in genetic diversity and population structure analysis in cymbidiums
Source: BMC Genet. 2014 Dec 5;15:124. doi: 10.1186/s12863-014-0124-5 (PMC4276258; doi:10.1186/s12863-014-0124-5)

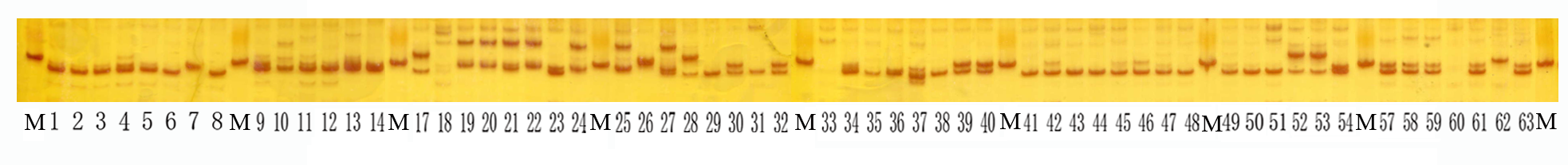


**a**

**b**

200


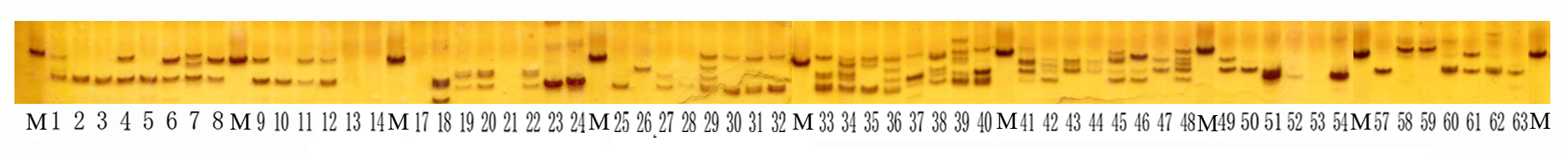


200

Supplement: Additional file 1: Figure S1. — Polyacrylamide gel electrophoresis profile of SSR62 a and SSR73 b. M: Maker DL2000; 1–63: cymbidium accession listed in Table 1. [file 12863_2014_124_MOESM1_ESM.doc]
